# Cross-Family Translational Genomics of Abiotic Stress-Responsive Genes between Arabidopsis and *Medicago truncatula*

**DOI:** 10.1371/journal.pone.0091721

**Published:** 2014-03-27

**Authors:** Daejin Hyung, Chaeyoung Lee, Jin-Hyun Kim, Dongwoon Yoo, Young-Su Seo, Soon-Chun Jeong, Jai-Heon Lee, Youngsoo Chung, Ki-Hong Jung, Douglas R. Cook, Hong-kyu Choi

**Affiliations:** 1 Department of Computer Science, Dong-A University, Busan, Republic of Korea; 2 Department of Medical Bioscience, Dong-A University, Busan, Republic of Korea; 3 Department of Genetic Engineering, Dong-A University, Busan, Republic of Korea; 4 Department of Microbiology, Busan National University, Busan, Republic of Korea; 5 Bio-Evaluation Center, Korea Research Institute of Bioscience and Biotechnology, Cheongwon, Republic of Korea; 6 Department of Plant Molecular Systems Biotechnology & Graduate School of Biotechnology, Kyunghee University, Yongin, Republic of Korea; 7 Department of Plant Pathology, University of California Davis, Davis, California, United States of America; University of Florida, United States of America

## Abstract

Cross-species translation of genomic information may play a pivotal role in applying biological knowledge gained from relatively simple model system to other less studied, but related, genomes. The information of abiotic stress (ABS)-responsive genes in Arabidopsis was identified and translated into the legume model system, *Medicago truncatula*. Various data resources, such as TAIR/AtGI DB, expression profiles and literatures, were used to build a genome-wide list of ABS genes. tBlastX/BlastP similarity search tools and manual inspection of alignments were used to identify orthologous genes between the two genomes. A total of 1,377 genes were finally collected and classified into 18 functional criteria of gene ontology (GO). The data analysis according to the expression cues showed that there was substantial level of interaction among three major types (i.e., drought, salinity and cold stress) of abiotic stresses. In an attempt to translate the ABS genes between these two species, genomic locations for each gene were mapped using an in-house-developed comparative analysis platform. The comparative analysis revealed that fragmental colinearity, represented by only 37 synteny blocks, existed between Arabidopsis and *M. truncatula*. Based on the combination of E-value and alignment remarks, estimated translation rate was 60.2% for this cross-family translation. As a prelude of the functional comparative genomic approaches, in-silico gene network/interactome analyses were conducted to predict key components in the ABS responses, and one of the sub-networks was integrated with corresponding comparative map. The results demonstrated that core members of the sub-network were well aligned with previously reported ABS regulatory networks. Taken together, the results indicate that network-based integrative approaches of comparative and functional genomics are important to interpret and translate genomic information for complex traits such as abiotic stresses.

## Introduction

Model systems may play a central role in translating genomic information from one species to another. A key rationale of sequencing the model species with enormous expenses is that knowledge gained from relatively small genome can be transferred to a related, but commonly larger and more complex, species and even across different families. Such cross-species translation is largely based on precisely identifying orthologous genes with shared evolutionary origins.

In plants, Arabidopsis is the first species whose genome was fully sequenced and contains perhaps the most comprehensive context of genomic information than any other model systems, and thereby can serve as the central model system for the translational genomics approaches. Comparative genomics should be the most prevalent analysis tool used for purposes of translating genomic information across different species, and works based upon the assumption that genomic contents and orders are, to some extent, conserved among different, but related, species despite of various levels of evolutionary changes. This assumption has been validated many times with respect to conservation of genomic structures and gene functions between orthologous loci. Naturally, the ability to transfer genomic information between different species depends on evolutionary distances and the nature of genomic change in particular regions. In the past, the translation of genomic information had, with limited capability, been conducted using common RFLP probes [1] and EST-based gene markers [2], [3]. Presently, it is becoming more direct, but even greater complex, due to strikingly rapid advancement of sequencing technologies (i.e., next generation sequencing or NGS) and resulting availability of genomic information. This technological advance leads the genomic science to a situation where the capability of bioinformatic analysis is increasingly emphasized.

Since the TAIR project (http://www.arabidopsis.org) first started in 1999, Arabidopsis has been playing a pivotal role, largely due to its well curated genomic information and functional annotation, in translational genomic studies within the Brassicaceae, including a broad range of related crop species such as *Brassica oleracea* (C genome, n = 9) [4], *B. rapa* (A genome, n = 10) and amphidiploid *B. napus* (AC genome, n = 19) [5]. Arabidopsis genomic information was also used to compare its genomic structure with other fully sequenced eudicot species including grape [6], poplar and papaya [7], [8], and even with far more distant monocot species, such as rice [9] and maize [10]. It was shown that Arabidopsis genome had only scant synteny with relatively distant monocot species [9], [10]. However, coexpression-based comparative network analysis of orthologous genes predicted between Arabidopsis and rice showed considerable level (approximately 77%) of conserved coexpression patterns [11], suggesting that cross-species translation of conserved functional genomic context would be more meaningful rather than simple structural genomic comparison.

The legume family includes a broad span of species, with roughly 20,000 species and 700 genera [12]. The family consists largely of three subfamilies: the Papilionoideae, with approximately 70% of species, which is a relatively recent-diverged group and includes most crop legumes; the Mimosoideae, with some 15%; and the Caesalpionoideae, known to contain the most ancient legume taxa, with the remainder. The potential of translational genomic analyses in the legume family and its application for the crop improvement may be particularly promising than any other plant groups, because well-defined model systems, such as *Medicago truncatula* and soybean, and a large number of domesticated legume species are available. More importantly, whole genome sequencing of five legume species, including *M. truncatula* [13], *Lotus japonicus* [14], *Glycine max* [15], *Phaseolus vulgaris* (unpublished, genome data available at Phytozome; www.phytozome.net) and *Cajanus cajan* [16], has recently been completed. At the dawn of the whole genome sequencing completion of these legume species, translational genomic approaches were suggested for the purposes of DNA marker development, positional cloning followed by gene discovery, and other omics studies with ultimate goals of legume crop improvement [17], [18]. As an example of the application, 5460 unigenes, which were identified as orthologs in *M. truncatula*, *L. japonicas* and soybean, were newly mapped on Pea (*Pisum sativum*) genome, and hence leading to comparative analysis of these four legume genomes [19].

Of these five legume species, *M. truncatula* should be a nodal legume model system that may have a potential as the translational interface to accelerate crop improvements in legumes, because of its evolutionary proximity and frequently found genome conservation with other economically important legumes [17]. Nevertheless, a functional genomic translation has yet not been conducted between these two representative model systems, Arabidopsis and *M. truncatula*. Therefore, as the first step toward cross-family translation of genomic information, it is expected that *M. truncatula*, due to its relatively better established genomic data than any other sequenced legume species and its diploid nature, will make it anchoring resource to translate genomic information from Arabidopsis, which is the representative model system of the Brassicaceae, into the legumes.

**Figure 1:**
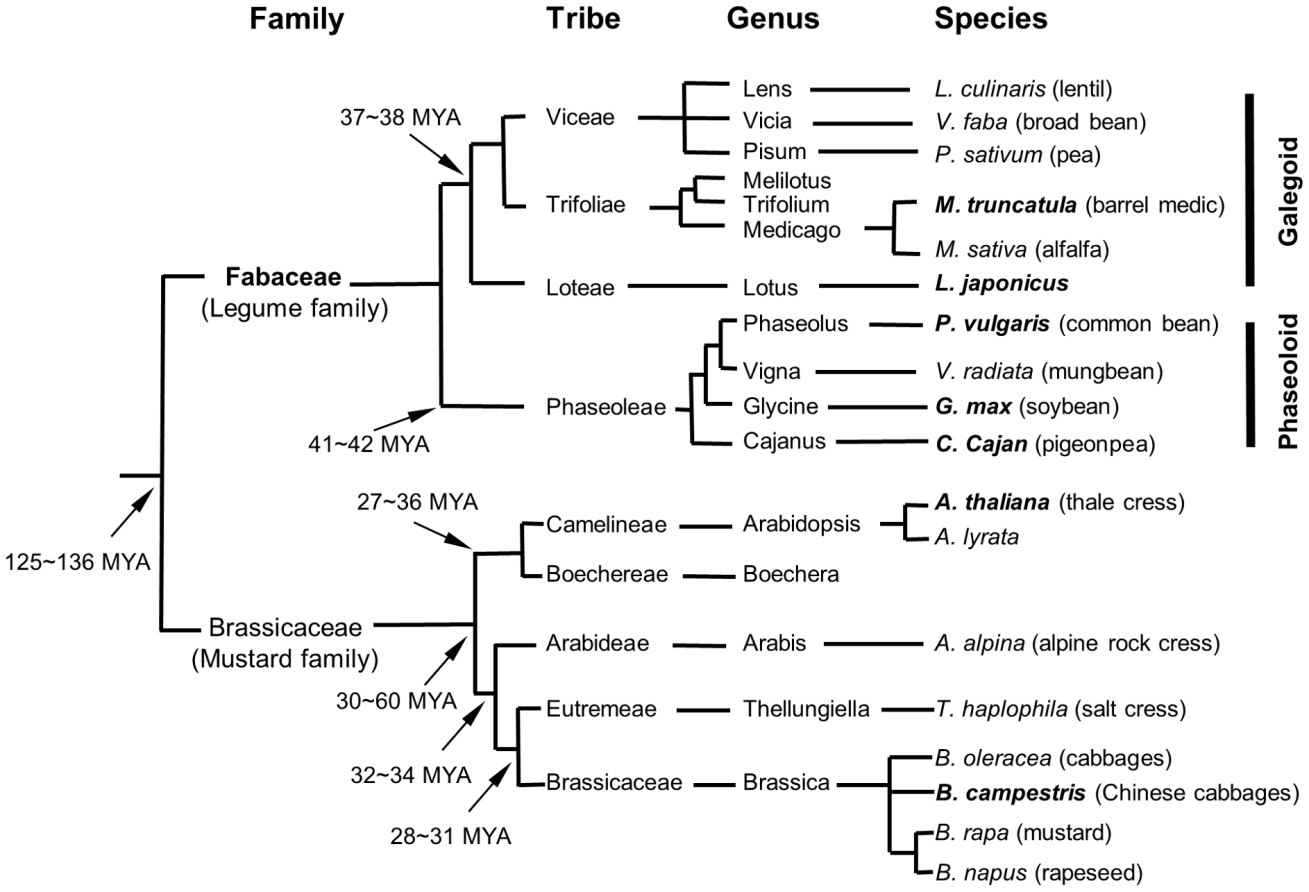
Taxonomic relationship of major crop and model species within two families, the Fabaceae and Brassicaceae. Whole genome-sequenced species are highlighted in bold italic. Most crop legumes occur in galegoid and phaseoloid clades. Known divergence time is denoted by arrows. MYA, million years ago.

These two families, the Brassicaceae and Fabaceae, in which contain significant portion of agro-economically important crops, are known to have diverged very long time ago, approximately 125-136 million years ago (MYA) [20], and presumably have experienced diverse scenario of evolutionary events (Figure 1). In addition to the ancient divergence time, even for model species with relatively small genomes, cross-species translation of genomic information is not malleable largely due to the complexity of genomic structures accumulated throughout the evolutionary history of given species.

In this study, we attempted to identify abiotic stress (ABS)-responsive genes, mainly using Arabidopsis as a central species for the translation of genomic information, and to transfer the genomic information into *M. truncatula*, as a anchoring model system within the legume family. Here, we report corresponding results, including genome-wide collection of ABS-related genes, estimation of cross-family translation rate, identification of key components in ABS response and genomic locus translation through comparative analysis. The main goal of this study is to provide researchers with basic information on genome-widely collected ABS-related genes towards the improvement of crop quality in ABS tolerance in the future.

## Materials and Methods

### Genome-wide screening of ABS-related genes and identification of cross-family orthologous genes

To search for the ABS-responsive genes, we employed different types of available data resources including literature, gene expression profiles, Arabidopsis database (TAIR at http://www.arabidopsis.org) and Arabidopsis Gene Index (AtGI; http://compbio.dfci.harvard. edu/tgi/cgi-bin/tgi/gimain.pl?gudb = arab). Genes associated with three major and the most important stresses (drought, salinity and cold stress) were screened in this study. In the events Arabidopsis DBs were used for the search, different key words relevant to the abiotic stresses were used to search ABS genes, and each of gene descriptions were investigated to confirm its functional relevance to the abiotic stresses. The genes collected from multiple data resources were combined into a single Excell worksheet and organized, basically, according to their locus IDs with other related information, such as annotations, expression cues, references and other related information (see Table S1).

For the first step of the translational analysis, the whole genome information for two model species, TAIR ver10 (http://www.arabidopsis.org) and Mt ver3.5 (*M. truncatula* HapMap Project; http://www.medicagohapmap.org/genome), was downloaded into our own server system. To identify orthologous genes between *M. truncatula* and Arabidopsis, we used in-house batch tBlastX/BlastP homolog search tools with the selected ABS-responsive gene set. Final decision on the orthology of pair-wise aligned genes was carefully made by taking integrated knowledge into consideration of the E-values, percent identities, cumulated total length of alignment and finally visual inspection for the quality of individual alignments.

### Genomic locus translation and comparative mapping

Comparative genomic mapping was conducted with the orthologous genes predicted between Arabidopsis and *M. truncatula*. Genomic locus positioning of each gene was made by using fully sequenced genomic information of Arabidopsis and *M. truncatula*. For purposes of the comparative analysis, we developed an in-house platform for the comparative mapping. Accordingly, ABS gene database was constructed to store and use the ABS gene information to produce the corresponding comparative maps with selected options, for example chromosome number selection and gene ontology criteria, for the analysis (the manuscript for the DB and analysis tool development will be prepared separately). Predicted orthologs were interlinked with other relevant information, such as functional description of genes, locus ID and expression cues, within the tentative orthologous gene database (TOGDB). To properly describe functional aspects of entire gene set, eighteen different gene ontology (GO) criteria were selected by considering appropriate level of GO hierarchies.

Synteny blocks were determined by following criteria: (1) each block must contain at least more than four consecutively collinear genes, (2) these collinear genes within the synteny block should have consistently increasing or decreasing locus IDs, (3) distance between neighboring genes should be less than 2Mb. Finally, the synteny blocks were determined by re-examining the orthologous relationships of genes in each block.

### Gene network and interactome analysis

To extract functional meaning of ABS-responsive genes and to connect this information with comparative genomic data, we employed a combination of tools and database including AraNet (http://www.functionalnet.org/aranet) [21] and CytoScape [22]. General gene network analysis pipeline was as follows. First, we used the web-based AraNet for the gene network analysis according to eighteen GO functional groups, and each of which gave ROC (Receiver Operating Characteristic) curve. Based on the ROC analyses, 12 GO criteria were further analyzed to select seed gene set. Initially, 125 seed genes were selected based on the evidence code (http://functionalnet.org/aranet/evidence_code.txt) and they were further analyzed against remaining GO groups, resulting in a total of 240 seed genes. The 240 gene set was further used to obtain additional protein-to-protein interaction (PPI) network data by integrating published PPI information [23], [24]. After removing overlapped portion of these two data sets, integrated PPI network was constructed, which was composed of 2,406 protein nodes and 6325 direct physical interactions. Sub-networks predicted to be associated with the abiotic stresses were further analyzed and selected from the integrated network by extracting gene and PPI networks shared between these two datasets. We further elaborated the selected sub-networks using the Cytoscape program and combined them with the information on seed genes and their corresponding phenotypes.

## Results

### Collection and identification of abiotic stress-responsive genes

**Figure 2:**
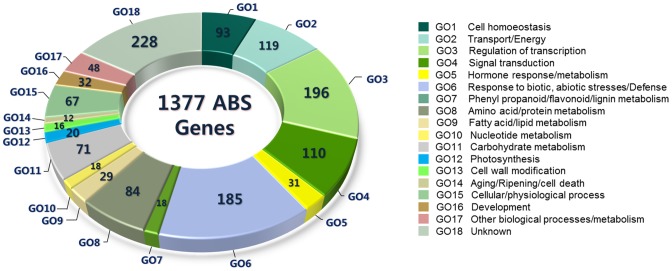
Classification of ABS-related genes according to 18 biological process gene ontology (GO) criteria.

To translate ABS-responsive genes across legume species, we used Arabidopsis as the central anchor model system, because of its richest contents of gene information among other plant model systems, to connect corresponding information with *M. truncatula*, the representative model legume species. In addition, dought stress-responsive genes identified by Affymetrix DNA microarray experiment in Grapevine (*Vitis vinifera*) [25] were translated into Arabidopsis orthologous genes, with the intent of initially centralizing all corresponding information into a single model system. TAIR database and AtGI were used as major resources for identification of ABS-related genes. Promoter-targeted data were also included [26]. As a result, a total of 1377 genes (1.4K gene set) associated with abiotic stresses were finally collected (Table S1) and classified according to the gene ontology (GO) biological process criteria. Eighteen GO terms were carefully selected to best describe the functional criteria of collected gene set, including cell homeostasis, regulation of transcription, signal transduction and so forth (Figure 2). Of these GO criteria, it is noticeable that stress/defense-responsive genes (GO6) took a relatively large portion, approximately 13.4%, of entire GO-based classification of the genes. In addition, transcription factors (GO3, 196 genes) and signal transduction-involved proteins (GO4, 110 genes), whose functions generally act at higher level of regulation in cellular process and may be collectively called transcriptional regulator proteins, occupied 22.2% in total, which is a largest portion amongst all the GO groups. This group of genes may act as central modulators in adaptation under ever-changing environmental conditions and the result indicates that many of them should significantly be involved in responses to the abiotic stresses. The MYB family proteins (55 genes) predominated the GO3 followed by zinc finger (31 genes) and NAC (NAM/ATAF/CUC, 14 genes) transcription factor family proteins (Table S1 & S2). Many of them contained genes previously well-known as responsive to abiotic stresses, of which, for example, included DREB/CBF (dehydration-responsive element-binding/C-repeat and DRE binding factor, 13 genes), AREB or ABF/ABI (ABA-responsive element-binding/ABA-insensitive protein, 5 genes), ANAC019 (At1g52890; positive regulator of ABA signaling) [27], ICE1 (At3g26744; inducer of CBF expression 1) [28] and many other genes (Table S1).

**Figure 3:**
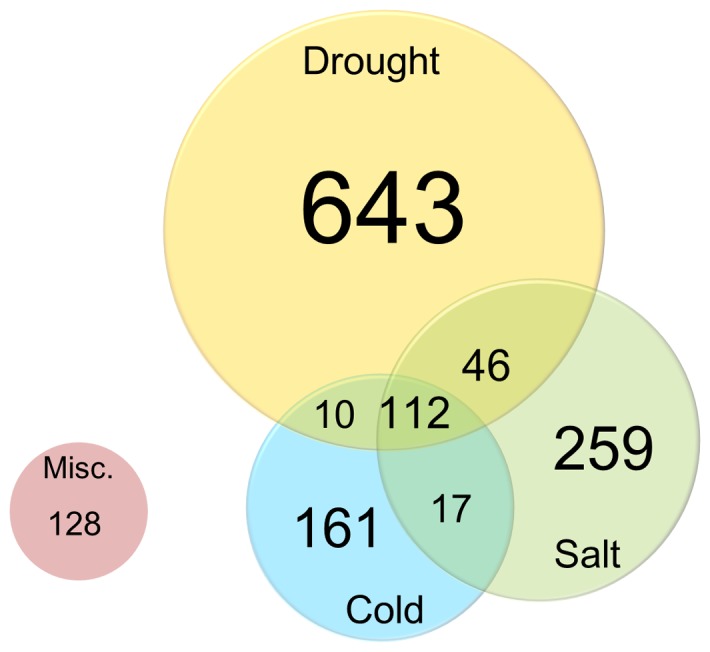
Distribution of ABS-responsive genes among three representative abiotic stresses, drought, cold and salinity. Expression cues for individual genes were determined by experimental platforms of relevant literature and the description of corresponding genes in the Arabidopsis database. Genes in the miscellaneous category usually had broad or ambiguous functional descriptions, such as abiotic stresses, ROS-responsive and ABA-induced.

To see how much portion of genes operate within the interface of different types of abiotic stresses or share their pathways, the 1.4K gene set was analyzed according to their expression cues (Figure 3). Out of three representative abiotic stresses, drought assumed the largest portion with a total of 811 genes followed by salt (434 genes) and cold stresses (300 genes). 128 Genes in the miscellaneous category include some indistinct, but potentially implicated with ABS response, descriptions for the expression cues to be classified into any of three criteria, such as “ABA-responsive” and “ROS-induced”, implying that they would be reclassified into any distinct groups in the future. It is noteworthy that a total of 185 genes appeared to respond against more than two different abiotic stresses. Of these genes, 112 genes seemed to respond in all the three different types of stresses, indicating that those genes and/or proteins involved in ABS responses share their pathways in certain interactive manner.

A total of twenty resources were employed to make the genome-wide collection of ABS-related genes, including 2 databases and 18 research articles (Table S1). Within the 1.4K gene set, 188 genes were hit by more than two references, which may implicate their functional involvement in ABS responses. These genes were further organized according to their GO (Figure S1). Interestingly, genes belonging to three GO classes (i.e., GO3/4/6), collectively called proteins associated with expressional regulation (GO3 and GO4) in response to abiotic stresses (GO6), occupied almost the half portion (86 genes, 45.8%) of the multi-hit genes (Figure S1, Table S3), which provide a major portion, but not all, of genes involved in both upstream and downstream of the ABS-responsive gene networks. This gene set appears to contain many functionally important proteins, for example RD29A/LTI78 (At5g52310; responsive to dehydration/low temperature-induced; hit by 10 references), RD26/ANAC072 (At4g27410; hit by 8 references), RD20 (At2g33380), COR15a and COR15b (At2g42540 and At2g42530, respectively; cold-regulated proteins) and many others. Of those 86 genes, 22 genes were shown to be induced by all three abiotic stresses, in some cases even by pathogen infection (Table S3), reconfirming the fact, as already shown in the Figure 3, that drought/cold/salt stresses dynamically interact with each other and potentially even with pathogenic processes.

### Identification of cross-family orthologous genes and translational efficiency of genomic information

**Figure 4:**
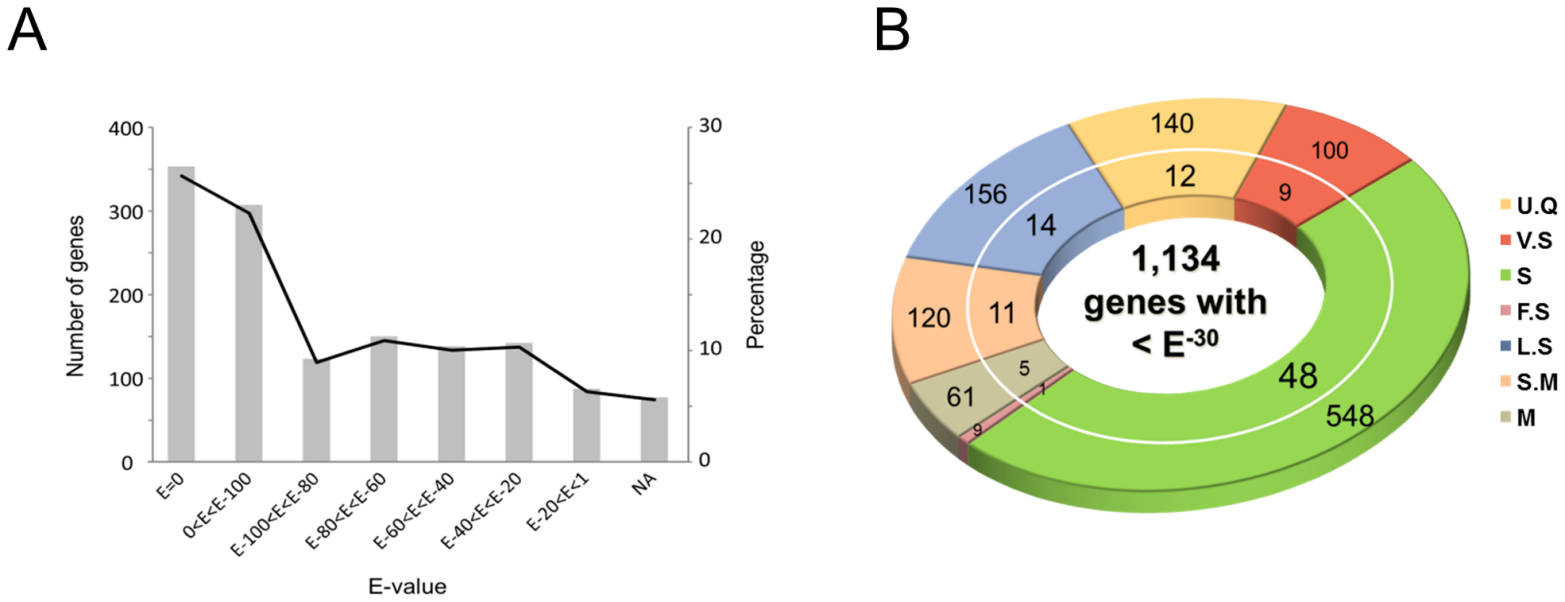
Cross-family translational efficiency of ABS-responsive gens between Arabidopsis and *M. truncatula*. A. Prediction of cross-species orthologous genes according to the Expect values. Bars indicate the number of genes, while the solid lines the percentage of translated genes. B. Distribution of translated genes with the E-value of <E^−30^ according to visual inspection of the tBlastX alignments: UQ, unique; VS, very specific; S, specific; FS, fairly specific; LS, less specific; SM, specific but multiple homologs; M, multiple homologs. The numbers in outer circle denote the number of genes, while the numbers of inner circle denote corresponding percentage.

Accuracy of identifying orthologs within the evolutionary context is crucial in translating genomic information between different species. For purposes of cross-family transfer of genomic information, we used a combination of parameters and tools by taking an integrative consideration of E-value and bit-score (i.e., a total length of corresponding alignment) of the tBlastX/BlastP-derived alignments. Final decision for the cross-species orthology was carefully made by scrutinizing resulting alignments along with investigator's comments. Firstly, we divided the translated genes according to the E-values, because it is a fundamental parameter of the first priority to be considered for the orthologous gene prediction, with regular intervals (Figure 4A). Of these, 352 genes (25.6%) with the value of E = 0 took the largest portion followed by 307 genes with 0<E<E^-100^, 123 genes with E^-100^<E<E^-80^ and 150 genes with E^-80^<E<E^-60^ of E value intervals. Taken together, a total of 932 genes (67.7%), although other genes above these E value intervals were not necessarily excluded from the ortholog criteria, showed reasonably significant E value-based prediction of orthology. Occasionally, E value-based identification of orthologs, in some sense, by an automatic prediction through bioinformatics data processing might be prone to errors mainly due to the complexity of genome, especially such as protein families resulting from gene duplications and homeologous chromosomes by polyploidization. Thus, we employed an additional means to ensure more accurate identification of orthologs by remarking and/or giving comments on actual tBlastX alignment outcomes (see legend of Figure 4 for the detailed information of remarks). The remarks fall into nine criteria (Figure 4). With the alignment remarks, a total of 829 genes (60.2%) were predicted as tentative orthologs above the level of FS (fairly specific) homolog search criterion (data not shown). To more accurately confirm the orthology of homologous candidates, genes with the value of <E^-30^ and remarks of UC (uncertain)/NA (not available) were removed and reanalyzed according to the alignment remarks, thereby resulting in a total of 1134 genes (82.4% of 1.4K gene set; Figure 4B). Of these, a total of 797 genes (70.3% of 1134 genes; 57.9% of 1377 genes) were determined potentially to be orthologous between these two model systems, which seems reasonable for the cross-family prediction of orthologous genes. In addition, it is noteworthy that the removal of genes with less significant E-values substantially contributed to increasing the probability, which increased from 60.2% to 70.3%, of finding orthologous counterparts between these two species (Figure 4B). These analyses also showed that the removal of genes with the E-values of low significance did not greatly affected the orthology prediction of genes above FS level of the remarks, by which only 32 genes (3.8% of 829 genes) were excluded, while below this remark level, 78 genes (18.8% of 415 genes without UC/NA criteria) were removed (Figure 4B). This result indicates that proper use of the E-value criteria is obviously useful for the translation of genomic information, but it has limitations to the accuracy in identifying orthologous genes. Therefore, it is recommended that a combination of approaches, including the E-value, total length of alignment and up-close investigation of individual alignments, are necessary to ensure cross-species translation of genomic information. The calculated GO-based translational efficiencies appeared various according to different GOs, ranging approximately from 60% (GO 12 & 16) to 96% (GO11) (data not shown). However, it looked obvious that low translation rates were always seen in the case where the gene numbers of corresponding GO were low (for example GO12/16), which may be more sensitively affected by counting predicted orthologs. Taken together, this implicates that translation of genomic information is not significantly affected by different functional criteria of genes.

### Genomic positional translation and integration of the comparative map with a gene sub-network

**Figure 5:**
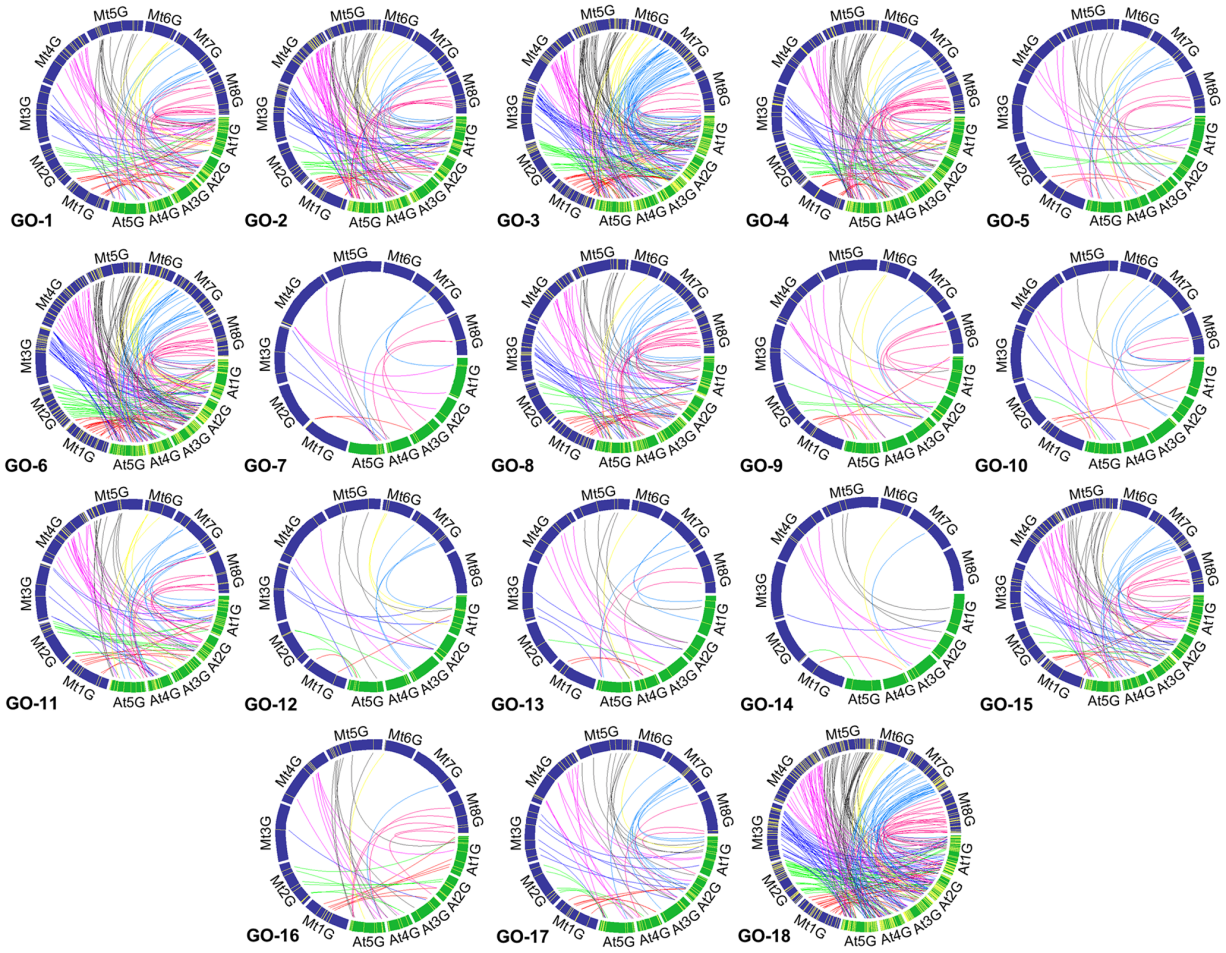
Cross-family locus translation of ABS-responsive genes through comparative analyses according to gene ontologies. Eight different colors, for *M. truncatula* chromosomes, were used to distinguish different chromosomal locations; Mt1G-red, Mt2G-green, Mt3G-deep blue, Mt4G-pale pink, Mt5G-black, Mt6G-yellow, Mt7G-sky blue, Mt8G-deep pink. At, *A. thaliana*; Mt, *M. truncatula*.

Comparative genomic analysis is an essential and efficient tool for translating genomic contexts of one species into other different, but taxonomically related, species. Comparative translation of genomic information is especially useful to save the research resources, not only because it enables to gain insights into genome-wide relationships among multiple genomes under comparison, but also because the translated information can be effectively applied to other organisms of researcher's interest. For purposes of interconnecting genomic information between Arabidopsis and *M. truncatula*, which represent two plant families of agricultural importance, loci of each predicted orthologous genes were positioned, based on the fully sequenced genomic locus information, in both model genomes. An in-house user-friendly platform for the comparative analysis was developed (relevant manuscript being prepared separately) and used to produce the comparative maps (Figure 5 and Figure S2).

Each of inferred orthologous gene loci were translated between the two genomes and analyzed for syntenic relationships. To avoid excessive complexity of resulting comparative maps, we conducted the analyses by translating locus information from single chromosome of one species to entire chromosomes of another compared species. Figure S2 A&B demonstrate Arabidopsis-centered comparison and Medicago-centered analysis, respectively. With 1377 gene loci, only 4.2% of 33,198 Arabidopsis annotated gene models [7], associated potentially with the ABS responses, we couldn't find extensive synteny blocks between these two species. The comparative analysis revealed only fragmental colinearity with a total of 37 synteny blocks (Figure S2 C, Table S4). The segmental syntenic relationship could readily be expected because two plant families of the Fabaceae and Brassicaceae, to which two model systems belong, diverged at the ancient time of approximately 125∼136 MYA (Figure 1) and consequently their genomes should have experienced a diverse array of evolutionary events. The numbers of synteny blocks on each Arabidopsis and *M. truncatula* chromosome ranged from 5 to 9 and from 2 to 9, respectively (Figure S2 A&B), while no synteny blocks were found in MtChr6 (Figure S2B). To see how genomic locations of ABS genes were correlated with their functional specialization, we counted ABS genes in each chromosome according to different GO criteria (Figure 5 and Table S5). Based on this analysis, although the data is not fully supportive, it seems, in general, unlikely that there is a significant correlation between the conserved synteny and functional organization of ABS-responsive genes within certain genomic regions. However, we could observe that relatively higher number of ABS genes resided in AtChr 1&5 and MtChr 4&5, which might implicate these genomic regions were relatively better equipped with ABS responses. Of these four chromosomes, interestingly, MtChr5 was most populated with ABS genes and showed the highest counts of ABS genes in the GO3/4/6 (Table S5), which are relatively more important functional criteria in the aspect of ABS responsiveness. On the other hand, it is interesting to note that any single synteny block was not found in the MtChr6, in which 52 ABS genes, relatively smaller number than in other chromosomes, were contained. This observation seems consistent with previous reports on comparative analysis done with limited number of genetic markers [29] and recent *M. truncatula* whole genome analysis [13] that MtChr6 is rich in heterochromatic DNA and relatively poor in expressed genes [12]. Furthermore, it has been known that the majority of NBS-LRR resistance analogous genes were localized as clusters in the MtChr6 [30], indicating that the MtChr6 is functionally specialized for the resistance mechanism against pathogen infections. Likewise, functionally specialized genomic regions for the ABS responses might exist and remain to be further elucidated with more extended data in the future.

A total of eight tandem duplication, two in Arabidopsis (ERD6 and glutathione S-transferase genes) and six in *M. truncatula* (ubiquitin-conjugating enzyme, sarcosine oxidase and six more), were found within the 37 synteny blocks (for detail see Table S4). On the other hand, genome-wide analyses of ABS genes resulted in identification of 49 tandemly duplicated genomic sites. Of these, 24 sites were tandemly duplicated only in Arabidopsis, 13 only in *M. truncatula* and 8 in both genomes (Table S6). It appears that genes tandemly duplicated in both genomes include mainly effector proteins by which exert their functions to recover the balance in homeostasis, with sufficient copy numbers, against the stresses. For example, these included glutathione S-transferase, plasma membrane intrinsic proteins (RD28), cytochrome P450 monooxygenase and many others (for more detail see Table S6). Interestingly, five copies of Na^+^/H^+^-exchanger, which is an important protein in regulating osmotic homeostasis, are shown to be tandemely duplicated in both MtChr4 and MtChr5.

**Figure 6:**
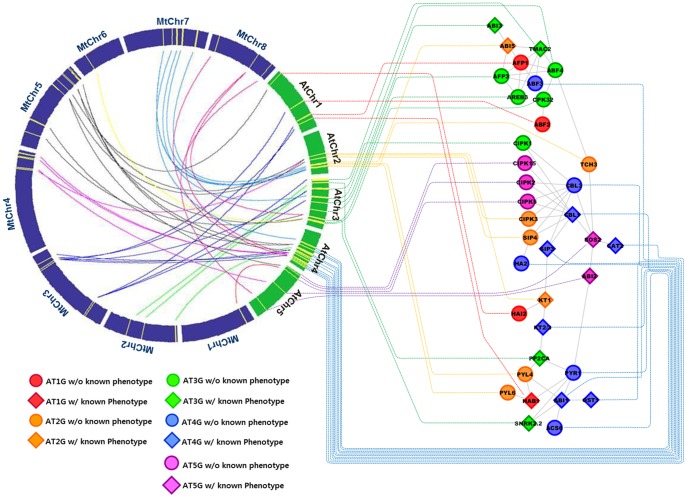
An example of integrative analysis for a core ABS regulatory sub-network and corresponding locus-translated comparative map. The sub-network was analyzed and reconstructed based on the Arabidopsis protein-to-protein interactome data. Availability of phenotypic information (for detail, see Table S7) and chromosome locations are denoted by a combination of different colors and shapes. Loci of individual genes within the sub-network are connected to corresponding positions on the Arabidopsis chromosomes. Detailed information of sub-network component genes is available in Table 1.

Function of genes involved in certain biological responses and/or metabolisms can be better triangulated when component genes are considered within the context of functional gene networks. Subsequently, as a prelude of the comparative functional genomic analysis, we attempted to integrate one of gene sub-networks, in which all interactions among genes were supported by physical protein-to-protein interactions [23], with corresponding comparative map (Figure 6).

The sub-network presented in Figure 6 elucidates a central ABA-mediated pathway in response to abiotic stresses, in which harbors mostly known core components of the pathway from the upstream, for example soluble ABA receptors (PYR/PYL/RCAR: pyrabactin resistance/PYR-like protein/regulatory component of ABA receptor), to the downstream, for example, two potassium channels (KT1 & KT2-3). Also, the network demonstrates correlations and/or connections between these components, including a series of cellular processes occurring upon stress such as perception, signal transduction, transcriptional regulation and expression of effector genes. The sub-network is composed of a total of 36 genes and can be further classified three groups: (i) perception and signaling-related sub-network; three ABA receptors, two SnRK2s (subfamily 2 snf1-related kinase), five PP2Cs (type 2C protein phosphatase); (ii) calcium-mediated signal transduction; one CaM (calmodulin), two CBLs (calcineurin B-like proteins) and nine CIPKs (CBL-interacting serine/threonine protein kinase); (iii) transcriptional regulation; six ABRE/ABF bZIP transcription factors and three AFPs (ABI five binding proteins, also known as Ninja-family protein, by which act as a negative regulator of bZIP TFs). In addition, five downstream effector genes are connected with the network: two potassium channels (KT1 & KT2-3), catalase (CAT2), H^+^-ATPase (HA2) and 1-amiocyclopropane-1-carboxylase synthase (ACS6; involved in ethylene synthesis) (Table 1, Table S7).

**Table 1:** List of genes involved in responses to abiotic stresses within the subnetwork.

	OrthologousLocusID		E-value	GeneSymbol	Annotation	Phenotype	Reference
	*Arabidopsis*	*M.truncatula*					
**AtChr1**	At1g07430	Mt5g080680	9.00E-108	HAI2	HighlyABA-inducedPP2Cgene2	NA	
	At1g45249	Mt3g101780	2.00E-56	ABF2	ABA-insensitive5-likeprotein	NA	
	At1g69260	Mt3g025970	1.00E-32	AFP1	ABIfivebindingprotein	NA	
	At1g72770	Mt3g104710	1.00E-118	HAB1	Proteinphosphatase2C16;homologytoABI1	Yes	N/A
**AtChr2**	At2g26650	Mt4g113530	0	KT1	PotassiumchannelAKT1	Yes	[52]
	At2g26980	Mt5g088350	0	CIPK3	CBL-interactingserine/threonine-proteinkinase3	NA	
	At2g30360	Mt5g075100	4.00E-154	SIP4	CBL-interactingserine/threonine-proteinkinase11	NA	
	At2g36270	Mt7g104480	3.00E-93	ABI5	ABAinsensitive5	Yes	[53]
	At2g38310	Mt7g070050	2.00E-73	PYL4	AbscisicacidreceptorPYL4	NA	
	At2g40330	Mt3g071740	3.00E-60	PYL6	AbscisicacidreceptorPYL6	NA	
	At2g41100	Mt7g087610	2.00E-51	TCH3	Calmodulin-likeprotein12	NA	
**AtChr3**	At3g02140	Mt3g025970	7.00E-07	TMAC2	Ninja-familyproteinAFP4;ABIfivebindingprotein4	Yes	[40]
	At3g11410	Mt2g066110	0	PP2CA	Proteinphosphatase2C37	Yes	N/A
	At3g17510	Mt2g045470	2.00E-159	CIPK1	CBL-interactingserine/threonine-proteinkinase1	NA	
	At3g19290	Mt3g101780	1.00E-67	ABF4	ABA-insensitive5-likeprotein7	NA	
	At3g24650	Mt7g083700	3.00E-36	ABI3	B3domain-containingtranscriptionfactorABI3	Yes	[54], [55]
	At3g29575	Mt3g025970	8.00E-31	AFP3	Ninja-familyproteinAFP3	NA	
	At3g50500	Mt8g079560	0	SnRK2.2	Serine/threonine-proteinkinaseSRK2D	Yes	[56]
		Mt5g018050	0				
	At3g56850	Mt6g026800	3.00E-84	AREB3	ABA-insensitive5-likeprotein2	NA	
	At3g57530	Mt5g089320	2.00E-177	CPK32	Calcium-dependentproteinkinase32	NA	
**AtChr4**	At4g11280	Mt7g079080	0	ACS6	1-aminocyclopropane-1-carboxylatesynthase6	NA	
	At4g17615	Mt5g096420	1.00E-88	CBL1	CalcineurinB-likeprotein1	Yes	[57], [58]
	At4g17870	Mt5g030500	2.00E-78	PYR1	AbscisicacidreceptorPYR1	NA	
	At4g22200	Mt2g006870	0	KT2.3	PotassiumchannelAKT2.3	Yes	[59]
	At4g26080	Mt3g104710	2.00E-112	ABI1	ABAinsensitive1	Yes	[60], [61]
	At4g26570	Mt5g096420	2.00E-136	CBL3	CalcineurinB-likeprotein3	NA	
	At4g30190	Mt4g127710	0	HA2	H(+)-ATPase2	NA	
		Mt3g108800	0				
	At4g30960	Mt4g131060	0	SIP3	CBL-interactingserine/threonine-proteinkinase6	Yes	[62]
	At4g33950	Mt8g079560	0	OST1	Serine/threonine-proteinkinaseSRK2E	Yes	[50], [63]
	At4g34000	Mt3g101780	6.00E-73	ABF3	ABA-insensitive5-likeprotein6	NA	
	At4g35090	-	-	CAT2	Catalase2	Yes	[64], [65]
**AtChr5**	At5g01810	-	-	CIPK15	CBL-interactingserine/threonine-proteinkinase15	NA	
	At5g07070	Mt5g075060	0	CIPK2	CBL-interactingserine/threonine-proteinkinase2	NA	
	At5g10930	Mt1g013700	0	CIPK5	CBL-interactingserine/threonine-proteinkinase5	NA	
	At5g35410	Mt4g114670	0	SOS2	CBL-interactingserine/threonine-proteinkinase24	Yes	[66]
	At5g57050	Mt8g102550	1.00E-78	ABI2	Proteinphosphatase2C77	Yes	[61]

There have recently been two important breakthroughs in ABA signaling model: (i) the discovery of soluble ABA receptor [31], [32]; (ii) the identification of PP2C-SnRK complex, negative and positive regulators of ABA receptor respectively, as a downstream component of ABA receptor complex [33], [34]. Due to these two important findings in 2009, ABA signaling model has been dramatically figured out that the PYR/PYL/RCAR-PP2C-SnRK2 complex plays the central role in response to abiotic stresses, such as drought and salinity [35]. In Arabidopsis, a total of 14 PYR/PYL/RCAR ABA receptor homologs have been identified [35]. Of these, three PYR/PYL/RCAR proteins (At2g40330, At2g38310, At4g17870) are involved as members of the sub-network, in which the ABA receptors are shown to interact with three PP2Cs (At1g72770; known as HAB1 for homolog to ABI1, At3g11410, At4g26080; known as ABI1) and two SnRKs (At3g50500, At4g33950; also known as OST1 for open stomata 1) (Figure 6). This tripartite ABA signaling core complex is shown to associate with the calcium-mediated regulation of gene expression. With regard to this, CBL and CIPK interactions play a major role in the downstream signaling. In Arabidopsis, 10 CBLs and 26 CIPKs constitute a complicated interaction network with some extent of functional redundancy [36]. Of these, three Ca^2+^ sensors and eight CIPKs participated in the sub-network, in which a diverse array of interactions between sensors such as CBLs and CIPKs were apparent (Figure 6) and well documented in genetic studies of plant's responses to the salinity, for example SOS (salt overly sensitive) pathway [37]. The same research group reported that SOS2 (a type of CIPKs) forms a complex with SOS3 (a type of CBL proteins), and lead to activation of SOS1 (a Na^+^/H^+^ antiporter) important for ion homeostasis and salt tolerance. In this SOS pathway, ABI2 (a type of PP2C) act as a negative regulator by deactivating SOS2 or dephosphorylating SOS1 [38]. The network seems to fairly well represent the CBL-CIPK-PP2C interactions among genes involved in the Ca^2+^-mediated signaling, although their modes of action, such as activation and deactivation, are not denoted. Within the network, it seems likely that the secondary signal of calcium ion is transduced mainly through CPK32, a calcium-dependent protein kinase, to AREB/ABF bZIP transcription factors. It was experimentally verified that AtCPK32 is involved in Ca^2+^-mediated transduction of ABS signals by interacting with ABF4, a transcriptional regulator [39]. In addition to ABF4, five more transcription factors (i.e., ABF2/3, AREB3 and ABI3/5, which are all bZIP TFs except for ABI3) constitute positive regulatory part of the TF sub-network. In contrast, three negative regulators, which are named as AFP (i.e., ABI five binding proteins), take parts in the transcriptional regulatory network (Figure 6). It is currently believed that AFPs are needed to fine-tune stress responses by interacting with ABI5 and/or ABI5-related bZIP TFs [40].

On the other hand, ion transporters play crucial roles especially in maintaining ion homeostasis through osmoregulation. Among others, potassium ion is the most important and abundant cation, and subsequently its corresponding channels or transporters may essentially affect the responses to abiotic stresses. Two potassium channels/transporters (KTs: At2g26650, At4g22200) are involved in the gene network in conjunction with the CBL-CIPK signaling pathway (Figure 6). Interestingly, it has recently been suggested that CBLs and CIPKs may compete with each other to bind with the channel and act in antagonistic manner, in which CIPK is a positive modulator and vice versa, to modulate KT's activity [41]. It has also recently been validated that HA2 (At1g 07430; a ABA-induced and seed specific PP2C, also known as HONSU) directly interacts with KT1 and regulates seed dormancy by acting as a negative modulator of ABA signaling [42].

Taken together, component genes within the chosen PPI-supported subnetwork appear to be well aligned not only with previously known functional studies, but also with interactome data [23], offering a nice case study of network-based translational genomic approaches. This type of genomic translation integrated with functional gene networks may facilitate more accurate transfer of genomic information between different species and can be applied to systematic functional studies of genes.

## Discussion

Whole genome sequences and complete gene sets within the genomic contexts, which have recently become available in many plant species, can provide broad insights into how genes and genomic structures are organized and have changed during the processes of adaptation to fit ever changing environment. Therefore, it is expected that the translational genomics, if one properly utilizes such approaches, may play critical roles, based on genome-wide understanding, to leverage some key areas, such as basic plant biology and development of more efficient selection processes for molecular breeding, ultimately towards the crop improvement. Genomic responses to abiotic stress involve, in both quantitative and qualitative senses, complex traits, which are determined by interactions between multiple genes. In this study, we intended to establish and provide a genome-wide list of ABS-responsive genes for the purpose of building up a basic resource for the cross-species translational genomics, between Arabidopsis and *M. truncatula*, and further gaining insight into key components governing the ABS-responses in plants.

Validation of homologous gene's orthology is the prerequisite for the comparative genomic translation, because multi-copy genes, for example family proteins, may frequently lead to erroneous transfer of genomic information. However, accurate prediction of the gene's orthology between different species is not straightforward largely due to complex history of speciation and duplication. A substantial effort has been made to transfer orthologous functional annotation of genes between different genomes. For example, EnsemblCompara Gene Trees [43], specialized only for vertebrates, and InParanoid [44], [45], developed mainly for eukaryotic species, employed phylogenetic approaches to resolve the complexity of ortholog identification, using TreeBeST method and two-pass BLAST, respectively. Particularly in plants, Arabidopsis played a central role in organizing functional annotation of genes in association with the Gene Ontology Consortium, and each of annotations were defined by 11 evidence codes, for typical examples ISS for “inferred from sequence similarity” and IPI for “inferred from physical interaction” [46]. The functional annotations of the gene in corresponding TAIR database (http://www.arabidopsis.org) is being continuously updated by integrating experimental data and literature-based information. Phytozome (http://www.phytozome.net) is one of the representative comparative genomics platforms for plants [47]. The database contains information of 25 completed genomic sequences and employs the common BLAST or BLAT (BLAST-like alignment tool) to perform the similarity searches. In contrast, GreenPhylDB (http://greenphyl.cirad.fr) provides comparative analysis platform specialized only for two representative plant species, Arabidopsis and rice, with the emphasis on phylogenomic approach for the family proteins [48]. On the other hand, Abrouk et al. [7] emphasized three components to ensure orthologous relationships between genes: (i) comparison of inter-specific coding sequence, (ii) similarity based on cumulative identity percentage (CIP), (iii) relative length of the sequences represented by cumulative alignment length percentage (CALP).

Although we did not develop or use an automated ortholog detection tool equipped with mathematically programmed algorithms, we tried to meet above orthology criteria, and subsequently orthologous genes were finally determined by inspecting individual alignments derived from the tBlastX/BlastP with highly trained human eyes and integrated knowledge on similarity searches. We also used our own multi-layered denotation on the sequence alignments from “unique” (for clear orthologous relationship; i.e., one-to-one relationship) to “multiple” (for multi-gene family and/or obscure orthology) (Figure 4). We imagined that the denotation would be especially useful when fully sequenced genomes, because they represented entirety of genomic context, were compared to translate the genomic information. Taken together, reliable range of translation rate was estimated approximately 60% (Figure 4) between Arabidopsis and *M. truncatula*. This translation rate is seemingly low, but probably reasonable if one considers their divergence time of 125∼136 MYA (Figure 1). This estimation could be applied when researchers intend to transfer genomic information between two plant species with similar divergence time.

In nature, plants are simultaneously exposed to multiple stresses, including abiotic and biotic stresses, and the stresses often interact with each other. Although many genes known to be involved in the ABS responses have been identified and elucidated within the context of gene interaction network [49], little has, in reality, been known about how these genes are interwoven within the network and interact between different abiotic stresses. Thus it would be critical to discover key cross-stress interplayers towards full understanding of orchestrated networks composed of ABS genes. In this study, a total of 185 (13.4% of 1.4K gene set) genes were identified as potential candidates for such interplaying components (Figure 3), although they could not be further analyzed towards the end of interactome analysis due to lack of information. However, we expect that the area or number of genes within the interacting interface of these three abiotic stresses would probably increase as more data and experimental evidences accumulate. Intriguingly, several recent reports suggest that the ABA, previously known as a central plant hormone regulating ABS responses, also play important roles in the interaction between abiotic stresses and pathogenesis [49]. One interesting example was shown that ABA was also involved in the defense response by closing stomata upon pathogen attack [50], thereby implicating that ABA plays its role against pathogen infection in more direct way than previously expected. As seen, a broader array of interactions between multiple stresses, including ABS and even biotic stresses, is becoming an emerging topic in this field of researches. Therefore, further analyses towards this direction are in great demand to shed more light on comprehensive understanding of network-based ABS responses, even with biotic stress responses.

Because ABS responses are QTL traits, extremely complex and thereby numerous genes are involved, the list of 1377 genes collected in this study seems large enough to explain full scenario of responses against abiotic stresses. However, the gene set is probably heterogeneous, because some portion of genes are perhaps be involved in general physiological responses, which means that individual genes may have differential significance in their functions. Various functions of genes commonly constitute networks within the hierarchical context at different levels on which they mainly act in the courses of reactions against given external stimuli. Thus, it is important to triangulate key genes from the central networks within which govern corresponding responses. In order for the comparatively translated genomic data to be more valuable, functional genomic data need to be integrated. For purposes of integrating these two different types of data, gene network and/or interactome analyses with the full set of 1.4K genes are currently under way (relevant manuscript will be prepared separately).

In addition to the functional aspect of the sub-network (Figure 6), we could observe some features of genomic organization of the sub-network genes. For example, we could find a couple of regions in which genes are localized in relatively closer proximity: (i) CIPK2/5/15 in terminal region of AtChr5, (ii) CPK32, AREB3 and SnRK2.2 in AtChr3, (iii) PYL4/6, TCH3 and ABI5 in AtChr2, (iv) CIPK3, SIP4 and KT1 in AtChr2 (Figure 6 & Table 1). It would be interesting to see that the AtChr4 is most populated or organized with genes from the broadest functional criteria of the gene network, which might be suggestive of structural organization, to some extent, for the functional specialization in ABS responses. However, the idea seems premature, remain elusive and need to be further assessed with more integrated data of genomic organization in conjunction with more extended gene network analyses.

As exemplified in Figure 6, the sub-network for ABA-triggered stress responses, although reconstructed only by using bioinformatic tools and/or resources, appeared to be well aligned with previously reported protein-to-protein interaction data [23], [49], and may play a central role towards further expansion of the network by integrating with other pathways, for example ethylene pathway (e.g., ACS6 in the sub-network) and ROS pathway (e.g., CAT2 in the sub-network). The subnetwork-associated comparative translation of genomic information presented in this study (Figure 6) was only a small example as a prelude towards a genome scale functional comparative analysis. The 1.4K gene set-wide gene/interactome network analysis is currently underway with necessary update of data and/or information, and the results will be integrated with structural genomic data, in the near future, with an ultimate goal of gaining broader and deeper insights towards understanding the whole picture of complex interactions of genes involved in abiotic stresses. This way, one would be able to better identify key components associated with tolerance mechanisms against ABS-derived challenges and can apply the gained knowledge for the molecular breeding programs. However, species-to-species translation of genomic information may not be such a straightforward process, where the situation is more crucial for complex traits such as the abiotic stresses, that one need to take a careful caution to enhance accuracy and/or quality of the information transfer. In leguminous plants, for example, such genomic translation was tried to discover core genes responsive to salt stress, and successfully done using a combination of transcriptomic and metabolomics data with six Lotus species [51]. In this particular example, we need to notice that they utilized translational genomic approaches within only closely related model and crop species from the same genus. On the other hand, even cross eudicot-to-monocot genomic translation was conducted with 4,630 orthologous gene pairs identified between Arabidopsis and rice [11]. Corresponding results showed highly conserved coexpression pattern of 77%, consequently indicating a broad range of applicability of translational genomics, when properly applied to species of interest.

Translational genomics, from a practical point of view, seems likely still in its infancy. It still has considerable obstacles to be overcome, such as development of more accurate ortholog finding algorithms, comprehensive curation of functional genomic data and reliable integration of large scale omics data. Despite all these obstacles, it seems most likely that translational genomics should be a strong tool to overcome the barriers in translating genomic context among different species. Towards this direction, the data gained from translation of genomic information could be utilized to identify trait-associated genes, and subsequently exploit marker discovery and development for the purpose of molecular breeding.

Since ABS-related genomic information has been translated into the legume model system *M. truncatula* in this study, it would readily become possible to conduct pan-legume translation of genomic information on ABS genes across fully sequenced legume species. Until now, five legume species have been fully sequenced and their genomic information is available in public domains. This situation may provide a great opportunity for translating functional discoveries in model systems into many other legume crops, which are relatively poor in genomic information, through breeding programs and molecular biotechnology. Furthermore, similar approaches could be applied in other functional criteria, which are of great biological importance in legumes, such as symbiotic nitrogen fixation, seed maturation and physiology, and secondary metabolisms. However, the efficiency and/or predictability of the cross-species translational genomic applications remain to be further refined so that individual genes and/or gene networks would functionally work properly for the complex QTL-associated traits such as the abiotic stresses. Nevertheless, we anticipate that our study would provide rich information on ABS-responsive genes and potentially contribute to identifying key genes and developing trait-associated molecular genetic markers towards the improvement of important crop quality related to the abiotic stress tolerance.

## Supporting Information

Figure S1 **GO-based classification of genes hit by multiple resources.** Three GO classes (GO3/4/6), which occupy largest portion and are associated with transcriptional regulation and response to abiotic stresses, are demonstrated separately using pie chart.
(PPTX)

Figure S2 **Locus translation and synteny analysis between Arabidopsis and** ***M. truncatula*** **.** A. Comparative analyses of single Arabidopsis chromosome-to-*M. truncatula* whole genome. Each of five Arabidopsis chromosomes was separately, to avoid excessive complexity, analyzed with eight chromosomes of *M. truncatula*. Corresponding syntenic comparative analyses are shown side-by-side. B. Comparative analyses of single *M. truncatula* chromosome-to-Arabidopsis whole genome. Each of eight chromosomes of *M. truncatula* was separately analyzed with five Arabidopsis chromosomes. Corresponding syntenic comparative analyses are shown side-by-side. C. Integrated comparative map and synteny analysis of translated ABS-responsive gene loci.
(PPTX)

Table S1 **Genome-wide list of genes associated with abiotic stress responses.**
(XLSX)

Table S2 **Classification of transcription factors (GO3) and signaling-associated proteins (GO4).**
(XLSX)

Table S3 **List of GO3/GO4/GO6 genes hit by multiple resources.**
(XLSX)

Table S4 **Summary of genes identified within the synteny blocks.**
(XLSX)

Table S5 **Distribution of abiotic stress-responsive genes in chromosomes according to the GO functional criteria.**
(XLSX)

Table S6 **List of tandemly duplicated genes.**
(XLSX)

Table S7 **Phenotype information of genes within the sub-network.**
(XLSX)
